# Analytical Approaches for Analysis of Safety of Modern Food Packaging: A Review

**DOI:** 10.3390/molecules25030752

**Published:** 2020-02-10

**Authors:** Magdalena Wrona, Cristina Nerín

**Affiliations:** Department of Analytical Chemistry, Aragon Institute of Engineering Research I3A, University of Zaragoza, María de Luna, 3, 50018 Zaragoza, Spain; magdalenka.wrona@gmail.com

**Keywords:** food packaging, volatile compounds, non-volatile compounds, IAS, NIAS, analytical techniques, antioxidants, odorous compounds, nanoparticles, EU legislation

## Abstract

Nowadays, food packaging is a crucial tool for preserving food quality and has become an inseparable part of our daily life. Strong consumer demand and market trends enforce more advanced and creative forms of food packaging. New packaging development requires safety evaluations that always implicate the application of complex analytical methods. The present work reviews the development and application of new analytical methods for detection of possible food contaminants from the packaging origin on the quality and safety of fresh food. Among food contaminants migrants, set-off migrants from printing inks, polymer degradation products, and aromatic volatile compounds can be found that may compromise the safety and organoleptic properties of food. The list of possible chemical migrants is very wide and includes antioxidants, antimicrobials, intentionally added substances (IAS), non-intentionally added substances (NIAS), monomers, oligomers, and nanoparticles. All this information collected prior to the analysis will influence the type of analyzing samples and molecules (analytes) and therefore the selection of a convenient analytical method. Different analytical strategies will be discussed, including techniques for direct polymer analysis.

## 1. Introduction

For decades, traditional food packaging was associated with the idea of mere containers created to protect food products from the environment during transport and storage operations. Nevertheless, today’s needs cannot be met any longer by traditional packaging, and more advanced and creative forms of food packaging [1] are demanded.

Modern society requires modern solutions, especially in the area of food safety and thus food packaging. Active packaging is an innovative concept that was spread out during the last few years. It is a novel approach for extending the shelf-life of food products without compromising their qualities. Active packaging is based on the idea of packaging with a specific design that is able to exert a positive interaction with the food. Therefore, during the storage it is possible to prevent food from deterioration with simultaneous maintenance of its original sensory properties [2].

Active packaging is a system where an active agent is intentionally incorporated into a specially selected part of the packaging. This is an interactive technology, where an active compound can be freely released (into the head space of packaging or food) or it can act as an antioxidant without direct contact with food. There are also different investigations conducted on the controlled release of active compounds, where required concentrations in special conditions can be released. Moreover, removal of unwanted, damaging substances from food surroundings by absorption process is a well-known technology. All this requires the design of special technologies and application of different food packaging materials [3]. Packaging encompasses a wide range of material types across plastics, glass, metal, and paper. Due to their chemical structure and properties, the most applied materials in food industry are plastics. In addition to traditional petroleum-based polymers, biodegradable plastics and biopolymers can also be found today on the market. In second place, paper and cardboard can be found [4].

It should be highlighted that food contact materials (FCMs) can be very complex structures created with application of adhesives. Multilayers can not only be composed of polymers, but also paper and/or aluminum that are often coated by adhesives and printing inks.

All this extensive knowledge on types of technologies and materials applied in the packaging industry and novel scientific solutions such as active packaging let researchers perform investigations on packaging safety. 

## 2. Migration

Although food packaging is beneficial and was created to assure quality and safety of food, it may also transfer harmful chemicals into contained food by migration process. The migration phenomenon is based on mass transfer, which occurs due to equilibrium of all chemical systems. Consequently, it can have adverse effects on human health due to incorporation of chemical substances from packaging origin into food structure. The transfer of migrants into food is not the same for different types of polymeric materials [5]. Moreover, the spectrum of potential migrants is extremely wide and some of them can be exceptionally toxic. When the packaging material is in the rolls or the packaging itself is stored one inside of another (e.g., coffee cups), a phenomenon called set-off migration can occur [6]. In this case, compounds from the outer part of packaging migrate to the inner part and consequently migrate to food/food simulant [7]. Therefore, the investigation on migration of specific chemical compounds from food-packaging is of great importance. 

## 3. Legislation

Materials for food packaging available on the market needs to be safe for the health of consumers and are not allowed to cause any changes in composition of the packed products. Plastics especially must meet strict formal requirements. In Europe, it is regulated by Commission Regulation No 10/2011 [8] on plastic materials and articles intended to come into contact with food. This legislation specifies rules of migration experiments and also contains a list of substances, together with their specific limits (SML), that may be intentionally added to the plastic materials during its manufacture. It also indicates how to evaluate not listed substances (NLS). If the substances are not included in this list, their migration should not be higher than 0.01 mg/kg (ratio 6:1) of food or food simulant [9]. Moreover, for NLS, a procedure of toxicity determination can be provided by the threshold of toxicological concern (TTC), including classification of analytes to Cramer classes. TTC approach has been recommended by the European Food Safety Authority (EFSA) [10]. 

It needs to be highlighted that migration is commonly carried out using different food simulants: liquid (e.g., 95% ethanol, 3% acetic acid) and/or solid (Tenax^®^). Analytical methods applied for analysis of food simulants depend on their chemical properties. Examples of analytical methods for screening of volatile and non-volatile migrants in different food simulants can be seen in Table 1. Naturally, if target analysis is performed, other analytical methods such as gas chromatography coupled to flame ionization detector (GC-FID), high-performance liquid chromatography coupled to ultraviolet detector (HPLC-UV) and high-performance liquid chromatography coupled to fluorescence detector (HPLC-fluorescence), and ultra-high performance liquid chromatography coupled to triple quadrupole mass spectrometry (UPLC-QqQ-MS)—can be applied.

Finally, the research on the potential toxic compounds from plastic materials for food contact applications is still undertaken and the law is constantly being improved. Therefore, twelve amendments to Regulation (EU) No 10/2011 were published and consolidated with the principal document. Implemented changes include correction of the European Union list of authorized substances by adding new substances, as very often EFSA adopts a favorable scientific opinion on a possible extension of the use of some substance. Other changes were based on modifications of the conditions for use and migration limits for specific substances and setting food simulants to be used for testing of products based on acidity. An example can be the restriction of the use of bisphenol A in plastic infant feeding bottles. In addition, restrictions were made to the purity criteria of some substances.

## 4. Analytes

**Figure 1:**
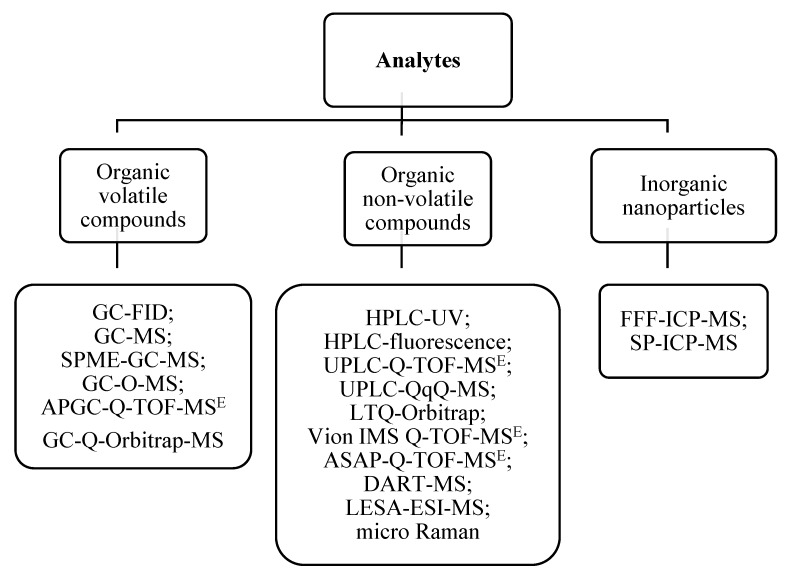
Dependency diagram of analytes from food contact materials and example of analytical methods that can be applied for their analysis. GC-MS—gas chromatography coupled to mass spectrometry; GC-O-MS—gas chromatography with olfactometry coupled to MS detector; APGC-Q-TOF-MS^E^—atmospheric pressure gas chromatography coupled to quadrupole-time of flight mass spectrometry^Elevated Energy^; GC-Q-Orbitrap-MS—gas chromatography coupled to quadrupole-Orbitrap mass spectrometry; LTQ-Orbitrap—hybrid linear ion trap-high resolution mass spectrometry combined with mass spectrometry; Vion IMS Q-TOF-MS^E^—Vion ion mobility quadruple time-of-flight with MS^E^ technology; ASAP-Q-TOF-MS—atmospheric solids analysis probe coupled to quadruple time-of-flight with MS^E^ technology; DART-MS—direct analysis in-real-time coupled to mass detector; LESA-nESI-MS—liquid extraction surface analysis nano-electrospray mass spectrometry; FFF-ICP-MS—field-flow fractionation coupled to inductively coupled plasma mass spectrometry; SP-IC-MS—single particle mode coupled to inductively coupled plasma mass spectrometry.

In general, among food contaminants we can distinguish migrants, set-off migrants from printing inks, polymer degradation products, and volatile compounds that may compromise either human health or the organoleptic properties of food. All mentioned phenomena involve the presence of molecules. Therefore, those compounds should first be identified, then quantified, and finally their toxicity should be determined. The list of possible chemical migrants is very wide and includes antioxidants, antimicrobials, intentionally added substances (IAS), non-intentionally added substances (NIAS), monomers, oligomers, and nanoparticles. Here, the most interesting and challenging cases from the analytical point of view are discussed. Figure 1 presents the general classes of analytes from FCMs and examples of possible analytical methods applied for their analysis.

### 4.1. Non-Intentionally Added Substances

Noni-ntentionally added substances (NIAS) are chemicals that are present in food contact materials but were not added to the polymer structure during its manufacturing; nonetheless, they can be detected in food simulants during migration assay. The origin of NIAS can be very different and the following substances can be classified as NIAS: (a) impurities from chemicals or primary materials; (b) contaminants in the case of recycled materials; (c) degradation products from polymer matrix and polymer additives; (d) products of reaction between polymer components or reaction of polymer with food [24,25]. Table 2 presents some NIAS detected in real samples of food packaging materials. The analytical method applied to the analysis of each compounds is also indicated.

Analysis of NIAS is a very challenging task. In the case of already studied NIAS compounds, target-chemical analysis can be applied, where mass spectra and retention time is already known. While for new unknown NIAS, nontarget analysis is required, where analytical screening is applied with different approaches to determine all possible migrants. To do this, high-resolution mass spectrometry (HR-MS) techniques are required. They are described in Section 5. The qualitative analysis of NIAS is very difficult due to the lack of information on composition of material additives and puzzling chromatographic spectrum results. Commonly, NIAS are new, unknown compounds that have not been described in literature. In addition, there is a lack of analytical standards, which are highly demanded for confirmation of compounds [24,33].

Finally, from the point of view of EU legislation, NIAS compounds are very often not listed in the European Union list of authorized substances from Regulation (EU) No 10/2011. Therefore, their SML should be established by Cramer classification and TTC [10,34].

### 4.2. Oligomers

One of the interesting types of NIAS are oligomers, either present in the polymer or produced during the degradation of polymers. Presence and possible migration of non-volatile oligomers can be characterized by chromatographic peaks with the same m/z interval step due to appearance of monomer units. Generally, migration of oligomers of diverse chemical structure can be observed with mass lower than 1000 Da. Oligomers do not only come from conventional polymers. The biodegradable polymers and biopolymers also have a series of oligomers migrating to the food.

**Figure 2:**
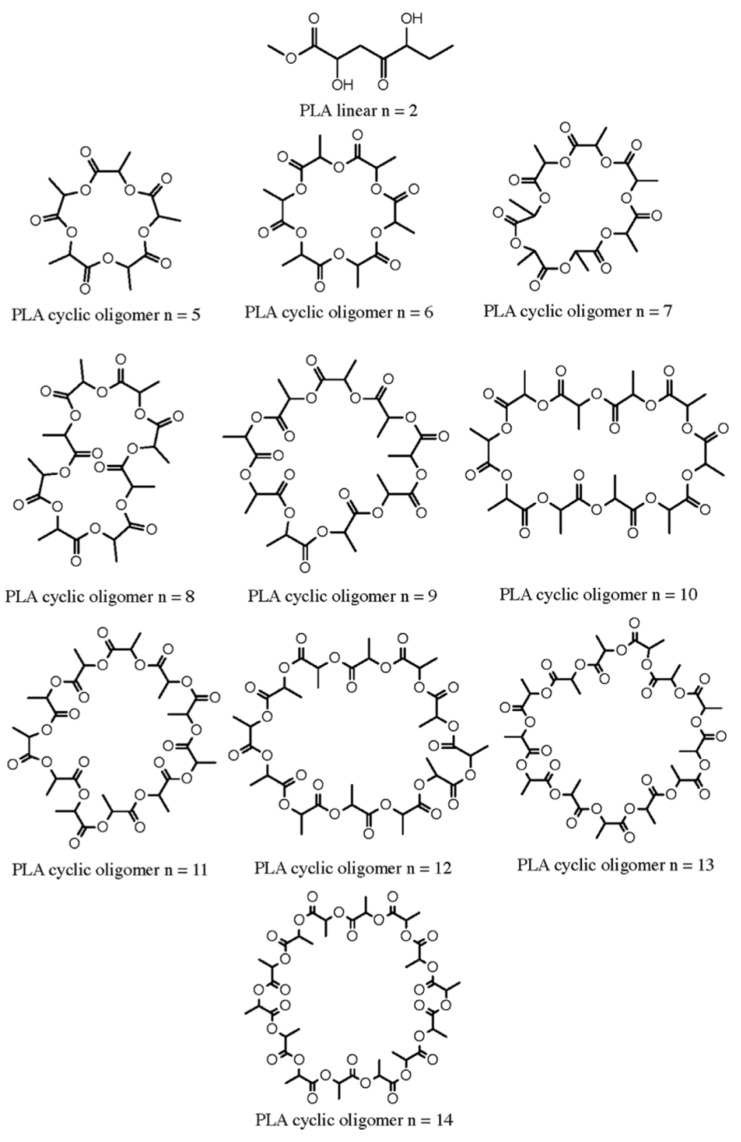
Structures of PLA oligomers. Reproduced from [15].

Different studies on migration of cyclic oligomers from biodegradable polylactic acid (PLA) and polyamide (PA) have been published [11,35]. PLA cyclic oligomers were found to be composed by lactic acid monomers and PA cyclic oligomers were composed by caprolactam monomers. Examples of structures of cyclic PLA oligomers detected as migrants from new active packaging are shown in Figure 2 [15]. Moreover, in the case of multilayer materials, oligomers from the adhesive used to build the different layers can be detected in the migration tests. Examples can be [PET/PU/PA/PU/CPP]_FCS_, where PET—polyethylene terephthalate; PU—polyurethane adhesive; PA—polyamide; and CPP—cast polypropylene. The food contact side (FCS)/food simulant contact side was CPP. In this case, complex oligomers such as cyclic esters consisted of phthalic acid (PA) and diethylene glycol (DEG) in different combinations were detected and identified [35]. All these migrants were analyzed by UPLC-Q-TOF-MS^E^—ultra-high performance liquid chromatography coupled to quadruple time-of-flight with MSE technology. In addition, oligomers from migration of PET, polyester, polystyrene (PS), polybutylene terephthalate (PBT), and polyethylene naphthenate (PEN) were described in literature [28,36,37,38].

It should be highlighted that, for quantitative analysis of oligomers, appropriate standards should be used. Unfortunately, up to now, most of the oligomers do not have commercially available standards. Some research groups obtained and purified some standards of cyclic esters and used them for quantitative purposes. However, in general, semi-quantification is applied using standards of oligomers that can be purchased. There is a lack of toxicity data of oligomers. So, it is recemented to apply the TTC procedure to evaluate its toxicity [12,36].

### 4.3. Antioxidants and Antimicrobials

Migration of compounds from food packaging polymer matrix can be influenced by incorporation of antioxidants. Therefore, antioxidant packaging not only protects food products but also can lower or totally prevent the migration of some compounds. An example is the reduction of migration of PLA oligomers in the case of new active packaging based on medicinal and aromatic plants powders [15].

When new active packaging is created, antioxidant or antimicrobial, the analysis of released compounds during the migration tests and risk assessment should be performed [3,39]. Not only the release of active agents, but also the potential impurities and compounds used to incorporate the active substances, should be under control. Obviously, this specific migration analysis should involve the screening analysis of all volatile and non-volatile compounds migrating to food simulants. One example is the migration of catechins and caffeine from active packaging with green tea for meat preservation. During the analysis by UPLC-Q-TOF-MS^E^, the main active agents were detected; but a further study in depth searching the individual catechins showed 9 different compounds in 10% ethanol and 95% ethanol. All of them were coming from green tea, which is food, and thus, accepted as active packaging, according to the Regulation 450/2009/EU. In this case, the used active agent is treated as foods bearing nutrition or health claims [40], and can be applied without any limits [41]. Other substances different from the active agents were not detected in specific migration analysis. Therefore, the analyzed active packaging met EU legislation for food contact materials. 

In the case of antimicrobial substances, some essential oils (EOs) have been demonstrated themselves as very efficient agents for active packaging applications [42,43,44,45,46]. Nevertheless, it was investigated and proved that micro-organisms from food can interact with EOs from antimicrobial packaging and produce new substances. As a result, bioconversion products such as methyl eugenol, styrene, and linalool oxide were formed from cinnamon essential oil by Aspergillus flavus strain [47]. Therefore, when applying analytical procedures for specific migration tests from antimicrobial packaging, the analysis of new substances coming from biotransformation should be taken into account. Moreover, those results show how important the application of migration assays from the new materials to real food is, and not only to food simulants. In this case, new analytical methods should be developed, as analysis of food is a difficult task due to complexity of the matrix.

### 4.4. Odorous Compounds

**Figure 3:**
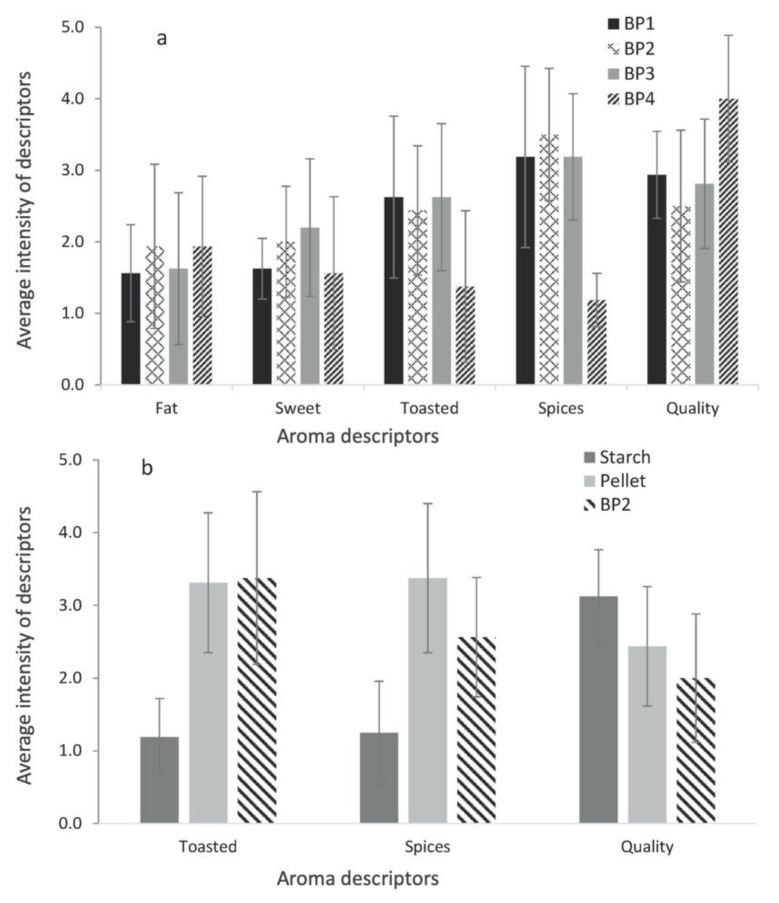
Bar chart of the sensory test for comparison between (**a**) 4 starch-based films (BP1, BP2, BP3—different starch-based polymers manufactured from starch powder provided by a Packaging Company; BP2—biopolymer manufactured from pellets provided by a Packaging Company; BP4—starch-based polymer from different origin) and (**b**) starch, pellets and film BP2. Reproduced from [49].

FCMs can be a source of odorous volatile compounds that can influence organoleptic properties such as taste and smell of stored food. It is especially important in the case of new FCMs such as biopolymers [30,48]. For this reason, it is important to study the aroma profiles of polymers, adhesives, printing inks, paper, cardboard and biopolymers, in order to be sure that they will not modify the sensory properties of food in contact with them. A quantitative descriptive analysis (QDA) should be performed to evaluate negative odorous attributes. One example is the evaluation of odorants from starch-based polymers by headspace solid phase microextraction gas chromatography with olfactometry coupled to mass detector (HS-SPME-GC-O-MS), where following analytes with their odor descriptors: trimethylamine (fish), 1-octen-3-one (mushroom), sotolon (spices, licorice), (*Z*)-nonenal (cucumber, fruit), (*E*)-nonenal (cucumber, green), eugenol (clove, honey), and p-vinyguiacol (clove, curry) were determined. They were the main compounds responsible for the aroma of starch-base films. Figure 3 shows the results of sensory evaluation and aroma descriptors of different samples of starch-based biopolymer [49].

## 5. Analytical Approaches

### 5.1. Volatile Compounds

Qualitative and quantitative analysis of volatile organic compounds (VOCs) in samples of migration assays is performed using GC–MS, APGC-Q-TOF-MS^E^, and GC-Q-Orbitrap-MS. These analytical techniques are highly required because it is necessary to use sensitive equipment capable of detecting analytes on the level of traces [50,51]. 

GC–MS typically uses electron impact ionization (EI), where analytes ionization is induced by 70 electron volt (eV) electrons. This ionization technique is considered harsh because of the high degree of molecules fragmentation. The application of the same operating conditions allows researchers to create databases containing mass spectra characteristic for each molecule. As a result, qualitative analysis of detected compounds is based on a search of spectral libraries [52]. 

Resolution power of GC-MS depends on the type of mass spectrometry (MS) analyzer. Time of flight instruments and Orbitrap are classified as high-resolution mass spectrometers (HRMS), where the molecular weights of analytes are determined to several decimal figures. It allows us to determine exact molecular formulas of unknown compounds. However, in the case of low-resolution mass spectrometers (LRMS), molecular weights of analytes are determined to the atomic mass unit. These types of instruments are more common due to lower prices, and easy and cheaper maintenance. On the other hand, HRMS can be applied as a complementary analytical technique to GC-MS. APGC is a soft ionization technique with less fragmentation of analyte. Here, energy of ionizing electrons can be modified and adjusted, therefore, the degree of fragmentation can be controlled. Reduced fragmentation results in higher sensitivity and selectivity. Identification of compounds is more difficult due to different operating conditions. However, if APGC is coupled to high-resolution MS, such as Q-TOF, the analytical tool is powerful enough to identify the chemical structure of the compounds using the chemical databases. It is also possible to create a homemade database with standards injected using different conditions. Another possibility is to use software provided by a company that matches possible chemical structures with the obtained spectra [30,53,54]. 

**Figure 4:**
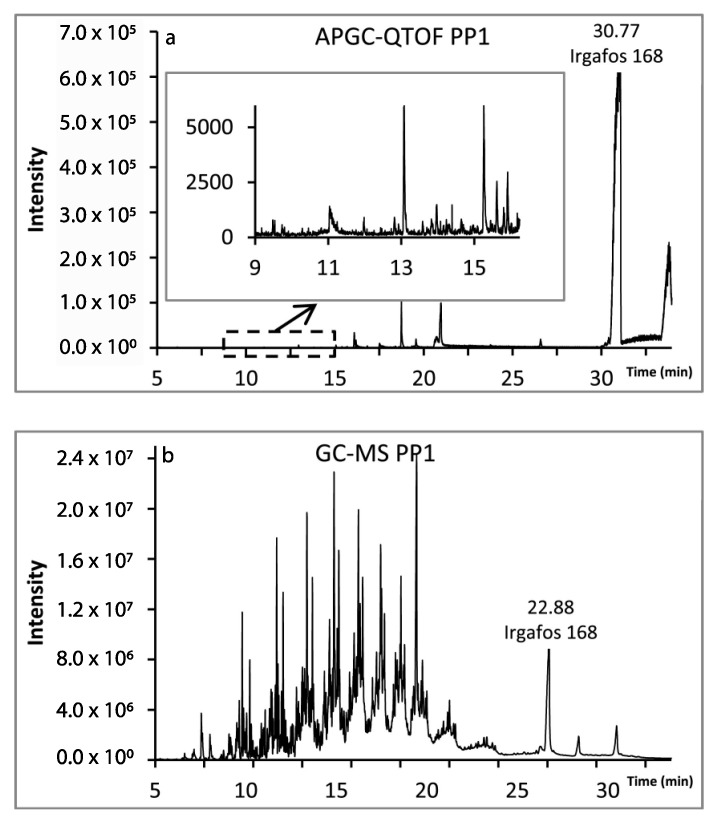
Chromatograms of polypropylene sample in (**a**) APGC-Q-TOF-MS and (**b**) GC-MS. Reproduced from [54].

Comparison of chromatograms obtained by GC-MS and APGC-Q-TOF-MS^E^ during nontarget screening of (semi)volatiles in food-grade polymers is shown in Figure 4 [54].

**Figure 5:**
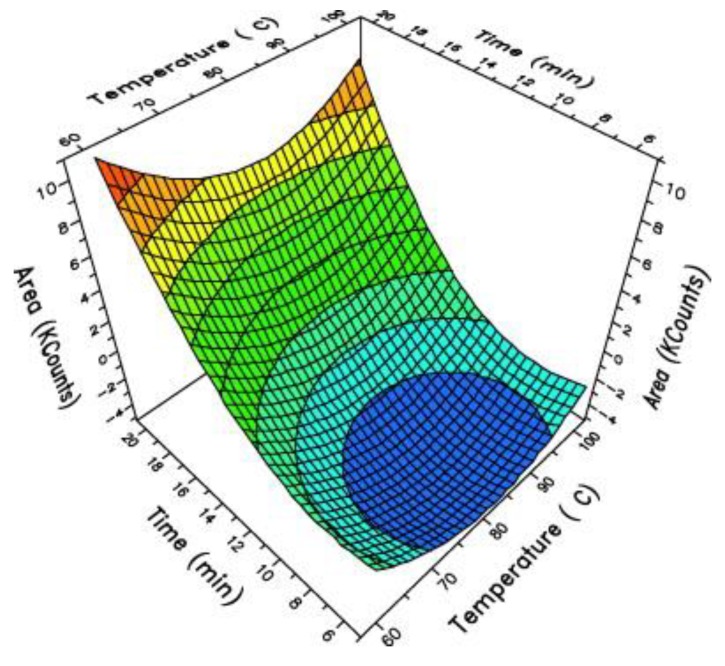
Surface contour plots from the optimization experimental set-up: effect of time and temperature of extraction over the total area counts. Reproduced from [57].

It should be highlighted that both GC-MS and high-resolution mass spectrometers commonly use liquid injection of samples such as, for example, the extract of dry food simulant or food simulants based on organic solvents. Moreover, sampling technique such as solid-phase microextraction (SPME) can be coupled to any commercial GC-MS system and allow it to increase sensitivity due to a high concentration rate on the microfiber. SPME is a solvent-free method, where the sample is absorbed or adsorbed on the sorbent—fused-silica-fiber-coated or chemically bonded with a specific extraction phase. After the sorption step, the fiber is directly injected into GC-MS. The type of sorbent is chosen according to the type of analyzed matrix (polarity and size of molecules). SPME offers the possibility of extraction of organic volatile compounds from aqueous samples (headspace and total immersion mode) and from organic solvents, even oil, only when a very small amount of sample is introduced into vial and headspace mode is applied. Moreover, SPME allows for direct analysis of solid samples such as polymer pellets, flakes, or plastic itself [55,56]. Obviously, in the case of SPME analysis, additional parameters of sample pretreatment (e.g., temperature and time of sorption) need to be set and optimized. Figure 5 shows an example where 3D response surface plots indicate the total peak area, considering temperature and time of extraction as independent variables for 1.0 g sample of PET pellets using 75 μm Carboxen^®^/Polydimethylsiloxane (CAR/PDMS) fiber for volatile compounds analysis. The optimum conditions are marked with red color [57].

An alternative to SPME can be Twister^®^, based on Stir Bar Sorptive Extraction (SBSE). It applies a magnetic stirring rod coated by a sorbent, which stirs aqueous samples, thus, sorption and concentration of VOCs is performed. After this, the Bar is transferred to either a Thermal Desorption Unit (TDU) or a Thermal Desorption System (TDS), where thermal desorption is carried out. After this process, the analytes are separated in GC system [58,59].

A very powerful technique for determination of migrants from active materials containing essential oils is the automatic multiple dynamic hollow fiber liquid-phase microextraction (HFLPME), where the extraction, preconcentration, separation, and cleaning are performed simultaneously using a semipermeable membrane. HFLPME is cheap, sensitive, and a versatile method, with the possibility of total automatization and miniaturization [60,61,62].

Another interesting tool for analysis of specific migration from food packaging is fabric phase sorptive extraction (FPSE). It is a new generation of sample preparation technique, where natural or synthetic fabric covered by ultra-thin coating sorbent is used as substrate. The exposure to an organic solvent or harsh chemical conditions (pH 1–13) does not affect FPSE, as is the case of other microextraction fibers. FPSE ensures high sorption area and selectivity. As any type of extraction solvent can be chosen, the sample can be analyzed not only by GC-MS but also by liquid chromatography [63,64]. 

Odorous compounds can be analyzed in any of described equipment. However, it requires the installation of a sniffing port that allows sensory detection by the human nose [65]. The combination of olfactometry with gas chromatography mass spectrometry (GC–O–MS) allows qualitative analysis of odorous compounds together with intensity evaluation of each odor and a descriptive analysis of the type of smell. Moreover, very often analytical detectors are much less sensitive than human odor perception registered by the odor panelists. It allows the detection of compounds with a very low odorous threshold that were not detected by MS [66,67].

### 5.2. Non-Volatile Compounds

The basic technique for the profiling of non-volatile compounds at very low concentrations in the samples from migration assay is liquid chromatography coupled to mass spectrometry (LC-MS). An intensive technology development in the field of chromatography let to design and develop different types of LC-MS interfaces. The chromatographic part of the instrumental equipment should provide good resolution of peaks, especially in the case of screening of nontarget non-volatile compounds from migration samples, where the number of different compounds simultaneously analyzed is usually very high. According to the analytical necessities, electrospray (ESI) ionization, atmospheric pressure chemical ionization (APCI), or matrix-assisted laser desorption-ionization (MALDI) can be applied [24,68].

**Figure 6:**
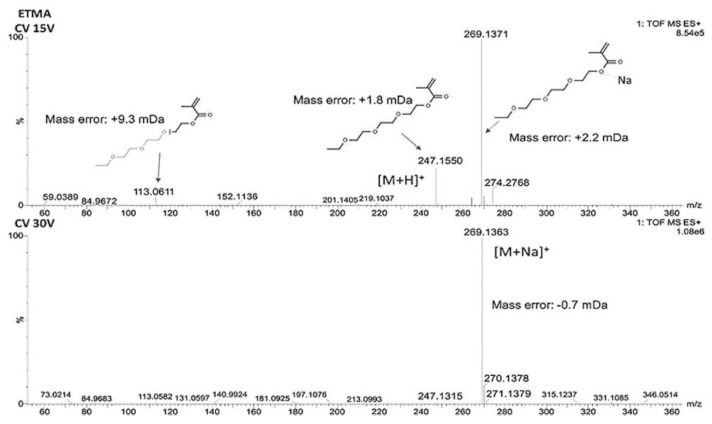
Identification of ethoxytriethylene glycol methacrylate in 50% ethanol using 15 V and 30 V cone voltages. Reproduced from [20].

High-resolution mass spectrometry techniques allow measuring the accurate mass of analytes and obtaining their elemental composition and, subsequently, their structure. Ultra-high performance liquid chromatography coupled to quadrupole-time of flight mass spectrometry^Elevated Energy^ (UPLC-Q-TOF-MS^E^) is a very attractive tool for nontarget analysis of non-volatile compounds from complex samples, because the obtained precursor and fragment ions have high sensitivity, high resolution, and high mass accuracy. MS^E^ is a patented acquisition mode, where low and high energy spectra are collected at the same time [27,69]. Figure 6 shows an example of spectra of ethoxytriethylene glycol methacrylate (ETMA), acquired using two different cone voltages (15 and 30 V) during the identification of non-volatile migrants from baby bottles by UPLC-Q-TOF-MS^E^ [20]. MS^E^ technology records data without necessity of its discrimination or preselection. As a result, each sample is completely catalogued in a single analysis. Comparing to standard MS or tandem MS-MS, MS^E^ detection is quicker.

A novel technology based on Ion Mobility Separation (IMS) together with Q-TOF-MS provides greater structural insight for the analysis of non-volatile compounds. IMS Q-TOF is able to resolve compounds that were coeluted and also permits the detection and identification of analytes covered by matrix or determines isomeric compounds that cannot be separated, applying standard chromatography or mass spectrometry. Moreover, the addition of the molecule shape parameter called collisional cross section (CCS), that is independent of the chromatographic conditions, permits the confirmation of the compounds by a comparison of the database of CCS values. CCS values are generated automatically for every single compound detected [70]. The application of this analytical technique permitted performing the design study and the identification of NIAS in UV varnishes for FCMs. The study highlights the advantages of ion mobility mass spectrometry for spectral interpretation and structure identification of unknown compounds. In this case, qualitative analysis has shown that some detected migrants in food simulants exceed the European food contact regulation limits [26].

Hybrid linear ion trap-high resolution mass spectrometry (LTQ-Orbitrap) combines a linear ion trap MS and the Orbitrap mass analyzer. It permits multiple levels of fragmentation (MSn) of analyzed compounds. Orbitrap provides high mass resolution and accuracy over a wide mass range—including the small molecule mass range. It has been already applied for qualitative analysis of non-volatile migrants from FCMs [31].

The analytical techniques described above are very powerful tools for nontarget analysis of non-volatile compounds. Nevertheless, in the case of target analysis connected with quantitative analysis, ultra-high performance liquid chromatography coupled to triple quadrupole mass spectrometry (UPLC-QqQ-MS) is a better option due to its very high sensitivity. Quantification of known non-volatile compounds in the migration samples can be done in two modes: single ion recording (SIR) or Multiple Reaction Monitoring (MRM). In SIR mode, only one specific *m*/*z* is collected, so it is applied when there is no suitable fragment ion to perform a more specific analysis; while MRM mode is a quick, highly sensitive, and selective method, where specific m/z and collision product ions are collected. An example of application of FCMs investigation is the determination of adhesive acrylates in recycled polyethylene terephthalate [64] or quantification of aromatic amines from polyurethane adhesives in food packaging by target analysis [17].

### 5.3. Surface Analysis

It is possible to perform direct analysis of FCMs (polymers) and solid food simulant (Tenax^®^) applying direct thermal desorption techniques such as atmospheric solids analysis probe (ASAP) MS, direct analysis in-real-time (DART) MS, desorption electrospray ionization (DESI) MS, liquid extraction surface analysis nano-electrospray mass spectrometry (LESA-nESI-MS), micro Raman, and surface enhanced Raman scattering (SERS). The mentioned techniques can be used as a first step of analysis of known compounds (target analysis), as they do not have separation steps. As a result, no sample handling is required, and the results are obtained quickly. They are highly recommended for target analysis and quick screening of the presence or absence of determined analytes. Some examples of application of direct analysis methods for polymers investigation can be (a) the fast assessment of oxo-biodegradable polyethylene film oxidation by SERS scattering with in situ formation of a silver nanoparticle substrate [71]; (b) direct screening of FCMs by LESA-nESI-MS [72]; (c) set-off of printing inks by DESI-MS-QTOF [73]; and (d) identification of nonvisible set-off in FCMs by DART [74].

### 5.4. Nanoparticles

Nanotechnology is expected to revolutionize and bring many areas to a higher level, and one of them is food packaging. Nanotechnology-enabled food packaging can help to extend shelf-life of packaged food and can also protect it against foodborne diseases. Moreover, the addition of nanoparticles into polymer matrix can improve its physical properties, such as barrier properties. Due to the increment of development of new FCMs containing nanoparticles, its safety should be constantly evaluating. This requires sensitive analytical techniques able to detect and quantify nanoparticles that can possibly migrate into food and food simulants [75]. The most common technique used for analysis of inorganic nanoparticles in solutions of food simulants is inductively coupled plasma mass spectrometry (ICP-MS). A single analysis can provide information about nanoparticles size and distribution and also the elemental composition. When applying ICP-MS, two different modes can be used: single particle mode (SP-ICP-MS) or ICP can be connected to the field-flow fractionation (FFF) technique to provide an additional separation step according to nanoparticles size. Moreover, the shape, size, and image of nanoparticles can be done using scanning electron microscopy (SEM), transmission electron microscopy (TEM), or atomic force microscopy (AFM). Nevertheless, these techniques have their limitations: at low concentrations of nanoparticles, which very often happens in case of migration assays, they have poor sensitivity [76].

## 6. Conclusions

The increased investigation on new packaging materials, enforced by market trends and consumer demand, results in the development of more complex FCMs, therefore, migration and food safety continue to be a very important topic. Nevertheless, comprehensive analysis of migration samples requires different analytical techniques as well as a wide knowledge about the types of technologies and materials applied in the packaging industry, the application of novel scientific solutions such as active packaging, and the type of potential migrants. All these compounds are molecules that should be qualified and quantified.

The most difficult group of compounds to identify in migration samples are NIAS, because they may be originated from different places and even be a product of side-reaction of polymer components. Moreover, the composition of new polymers and packaging is very often confidential, therefore, there is lack of information on materials additives. All of this makes the qualitative analysis a very challenging task. It would be highly demanded of the collaboration of chemical and plastic industries with the researchers working on plastic safety, in order to provide the necessary information about the chemicals’ and polymers’ impurities, additives, and possible side-reactions.

When applying the analytical procedures for migration tests of antimicrobial packaging analysis, new substances coming from biotransformation should be taken into account. It was shown that micro-organisms from food can interact with essential oils used as the active agent in antimicrobial packaging and can produce new substances as metabolites. Moreover, those results show how important the application is to real food, and not only using food simulants, for migration assays. In this case, new analytical methods should be developed, as the analysis of food is always a difficult task due to the complexity of the matrix.

From the analytical techniques point of view, advances in instrumental analysis and development and improvement of chemical databases would facilitate the quick detection of migrants. Currently, GC-MS, APGC-Q-TOF-MS^E^, and GC-Q-Orbitrap-MS are used for analysis of volatile compounds. In the case of new polymers, especially biopolymers, the analysis of odors should not be forgotten, due to their possible negative influence on the organoleptic properties of packaged food. Such analysis can be done by combination of olfactometry and gas chromatography; while complex analysis of non-volatile compounds is currently performed using high-resolution mass spectrometry techniques such as UPLC-Q-TOF-MS^E^, LTQ-Orbitrap, or UPLC-IMS Q-TOF-MS^E^. In the case of quantitative analysis, the application of UPLC-QqQ-MS is highly recommended due to its selectivity and sensitivity. Thermal desorption techniques for direct analysis of FCMs solid food simulant are also available. They can be used as the first screening step for determination of known and unknown volatile migrants as they are quick and do not require any sample handling. In the near future, the development of new analytical methods for nontarget analysis can be expected, using HRMS equipment to determine and quantify unknown migrants from food contact materials.

The introduction of nanotechnology to polymer science resulted in the creation of nanotechnology-enabled food packaging. It draws a need for evaluation of safety of polymers with nanoparticles. Therefore, in this specific case, sensitive analytical techniques able to detect and quantify nanoparticles should be used.

## Figures and Tables

**Table 1:** Examples of analytical methods for different simulants.

Food Simulant	Description	Analytical Method ^1^	Comments	Literature
**A**	Ethanol 10% (*v*/*v*)	SPME-GC-MS; UPLC-Q-TOF-MS^E^	Either by HS or total immersion modes	[11,12,13,14]
**B**	Acetic acid 3% (*v*/*v*)	SPME-GC-MS; UPLC-Q-TOF-MS^E^	Either by HS or total immersion modes	[11,15,16,17]
**C**	Ethanol 20% (*v*/*v*)	SPME-GC-MS; UPLC-Q-TOF-MS^E^	Either by HS or total immersion modes	[7,18,19]
**D1**	Ethanol 50% (*v*/*v*)	SPME-GC-MS; UPLC-Q-TOF-MS^E^	If SPME-GC-MS with total immersion of fiber is performed sample should be diluted at least 5 times.	[7,20,21]
**D2**	Any vegetable oil containing less than 1% unsaponifiable matter—can be replaced by 95% ethanol and isooctane	Liquid injection GC–MS; UPLC-Q-TOF-MS^E^ (reverse-phase phase column for 95% ethanol and normal-phase mode for isooctane)	If oil is used, it needs to be extracted. HS-SPME-GC-MS is also available for oil When using 95% ethanol and isooctane they can be concentrated under gentle stream of nitrogen to gain sensitivity.	[6,11,12,13]
**E**	poly(2,6-diphenyl-*p*-phenyleneoxide) known as Tenax^®^, particle size 60-80 mesh, pore size 200 nm	Liquid injection GC–MS; UPLC-Q-TOF-MS	Needs to be extracted with some organic solvent like for example ethanol or methanol and they can be concentrated under gentle stream of nitrogen to gain sensitivity. Three sequential extractions are usually applied	[6,22,23]

^1^ SPME-GC-MS—solid phase microextraction gas chromatography coupled to mass spectrometry (MS) detector; HS—headspace; UPLC-Q-TOF-MS—ultra-high performance liquid chromatography coupled to quadruple time-of-flight with MS^E^ technology.

**Table 2:** Example of NIAS detected in real samples of food packaging.

No	NIAS	Structure	Analytical Method ^1^	Packaging Material ^2^	Literature
1	2-propenoic acid, 1,1′-[2-[[3-[2,2-bis[[(1-oxo-2-propen-1-yl)oxy]methyl]butoxy]-1-oxopropoxy]methyl]-2-ethyl-1,3-propanediyl] ester	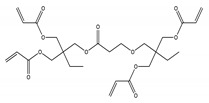	Vion IMS Q-TOF-MS^E^	PP varnished	[26]
2	2,6-toluenediamine	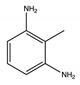	UPLC-Q-TOF-MS^E^	PU Ad	[17]
3	3,5-di-tert-butyl-4-hydroxybenzaldehyde	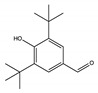	UPLC-Q-TOF-MS^E^	PP	[27]
4	bis(2-hydroxyethyl) phthalate	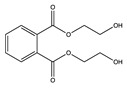	UHPLC-ESI-Q-TOF MS	PET/Al/PE	[28]
5	calcium carbonate	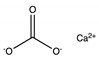	micro Raman	LDPE	[29]
6	mono-2-ethyloxoexyl adipate	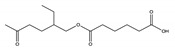	APGC-Q-TOF-MS^E^	PLA	[30]
7	*N*-[(9Z)-9-octadecen-1-yl]acetamide	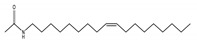	LC-Q-Orbitrap-MS	PLA	[31]
8	tert-butyl-1-oxaspiro (4,5) deca-6-9-diene-2,8-dione	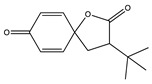	GC-MS	PE/PA	[32]
9	tripropylene glycol diacrylate	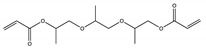	GC-Q-Orbitrap-MS	PLA	[31]

^1^ UHPLC-ESI-Q-TOF MS—ultra-high performance liquid chromatography with electrospray ionization coupled to quadruple time-of-flight with mass detector; LC-Q-Orbitrap-MS—liquid chromatography coupled to quadrupole-Orbitrap combined with mass spectrometry. ^2^ PU Ad—polyurethane adhesive; PP—polypropylene; LDPE—low-density polypropylene; PLA—polylactic acid; PET/Al/PE—polyethylene terephthalate/aluminium/polyethylene; PE/PA—polyethylene/polyamide.
